# Impact of Breast Cancer on Cardiometabolic Health in Spanish Women ≥50 Years with Pre-Existing Type 2 Diabetes Mellitus

**DOI:** 10.3390/cancers16162853

**Published:** 2024-08-15

**Authors:** Lucía Fernández-Arce, Nena Robles-Rodríguez, Ana Fernández-Feito, Rocío Fernández-Iglesias, María del Mar Fernández-Álvarez, Alberto Lana

**Affiliations:** 1 Department of Medicine, Faculty of Medicine and Health Sciences, University of Oviedo/ISPA, 33006 Oviedo, Spain; uo272621@uniovi.es (N.R.-R.); fernandezfana@uniovi.es (A.F.-F.); fernandezirocio@uniovi.es (R.F.-I.); lanaalberto@uniovi.es (A.L.); 2 Department of Surgery and Medical Surgical Specialities, Faculty of Medicine and Health Sciences, University of Oviedo/ISPA, 33006 Oviedo, Spain; fernandezmar@uniovi.es

**Keywords:** diabetes mellitus, type 2, glycated hemoglobin A, diabetes complications, breast neoplasms, cardiometabolic risk factors

## Abstract

**Simple Summary:**

Breast cancer and type 2 diabetes mellitus are highly prevalent and closely related diseases. Type 2 diabetes increases the risk of breast cancer. Our retrospective matched cohort study involving 318 women with type 2 diabetes assessed the evolution of cardiometabolic parameters and diabetic complications in women with (*n* = 106) and without subsequent breast cancer (*n* = 212). Over a 7-year follow-up period, women with breast cancer had worse glycemic control but not higher rates of diabetic complications compared to women without breast cancer.

**Abstract:**

During breast cancer (BC), cardiometabolic disorders can worsen prognosis, particularly in women with type 2 diabetes mellitus (T2DM). This study aimed to determine the impact of BC diagnosis on cardiometabolic parameters and the incidence of complication in women over 50 years of age (90% aged ≥ 65 years) with pre-existing T2DM. Using primary care registries from Asturias (Spain), a total of 106 women diagnosed with T2DM followed by BC were selected and matched with women with T2DM (*n* = 212) in a cohort study. Indicators of cardiometabolic health and microvascular complications associated with T2DM were collected. Women were monitored from two years prior to five years after BC diagnosis. Conditional logistic regressions were used to compare the adjusted odds of staying below each indicator’s threshold. During follow-up, women with T2DM+BC had a higher risk of fasting blood glucose ≥126 mg/dL (adjusted odds ratio [aOR] = 1.83; 95% confidence interval [CI95%]: 1.01–3.32) and glycosylated hemoglobin (Hb1Ac) ≥ 48 mmol/mol or 6.5% (aOR: 2.44; IC95%: 1.21–4.91). There was no difference between the groups regarding the incidence of microvascular complications. BC incidence negatively impacted the glycemic control of Spanish women with pre-existing T2DM measured by basal blood glucose and HbA1c, but not cardiometabolic health indicators or T2DM complications.

## 1. Introduction

There is strong evidence of an association between type 2 diabetes mellitus (T2DM) and an increased incidence of breast cancer (BC) [1,2,3,4], especially in Europe and North America [5,6]. Boyle et al. estimated that the risk of developing BC is increased by 27% in women with T2DM compared to women without T2DM (16% after adjusting for body weight) [1]. Several shared mechanisms and risk factors underlie this association, including the role of body weight and body fat [4,7]. However, other highly relevant and complex mechanisms exist, such as metabolic dysregulation resulting from dyslipidemia, hyperinsulinemia, hyperglycemia, hypoxic conditions, the inflammatory response, and sex hormone imbalances [8,9,10].

Additionally, pre-existing T2DM has been linked with more aggressive BC, as it is associated with a higher probability of more advanced stages, higher histological grade, larger tumor size, lymph node infiltration, and metastasis [8,11,12]. The mechanisms underlying the association between T2DM and BC also explain BC diagnosis with a worse prognosis after T2DM. In particular, sustained hyperglycemia may worsen BC prognosis by affecting tumor cell pathways as well as cell proliferation, the inhibition of apoptosis, migration, and invasion [13]. In addition, T2DM is also linked to a greater number of complications associated with BC treatment, increased vulnerability to infections, and overall worse health outcomes [14,15,16]. It is not surprising, therefore, that there is also evidence of an increased risk of dying when T2DM precedes a BC diagnosis. According to a meta-analysis by Zhou et al., a history of T2DM increases the risk of all-cause mortality in women with BC by 37% and the specific risk of BC mortality by 17% [3]. However, other recent studies caution that BC may be less responsible than expected for excess mortality [17,18]. Many women with prior T2DM die from cardiovascular events and other health problems not directly attributable to BC [19,20]. These findings suggest that a BC diagnosis in women with pre-existing T2DM leads to cardiometabolic disorders that complicate their prognosis [18]. Some studies found that hyperinsulinemia and chronic hyperglycemia, as measured by glycosylated hemoglobin (HbA1c) or fructosamine, were associated with poorer prognosis among women with BC [13,21,22,23,24]. However, because of the design characteristics, these studies could not establish whether metabolic disorders occurred as a consequence of BC or were related to previous T2DM.

Moreover, overlooking comorbidities during cancer treatment and follow-up is a source of concern for oncologists, as they could undermine the gains achieved through improvements in the early detection and treatment of cancer [25]. Considering the increasing frequency of T2DM and BC, understanding the systemic cardiometabolic effects that occur after the diagnosis and treatment of BC in women with T2DM is imperative [11,25,26]. Our study aimed to determine the impact of a BC diagnosis on the evolution of cardiometabolic parameters, including glycemic control, and the incidence of complications in women with pre-existing T2DM.

## 2. Materials and Methods

### 2.1. Study Design and Participants

A retrospective matched cohort study including women ≥50 years with a physician-confirmed T2DM diagnosis was conducted. This target population was selected using primary healthcare (PHC) clinical records from Asturias (Spain). Women who were assigned the ICD-10 diagnostic code E11 in their electronic medical records (which includes all forms of T2DM) on 1 January 2012 were selected for this study. In addition, women with a T2DM diagnosis contained in the description of their clinical episode were also included.

Data from these women were cross-referenced with information from a population-based cancer registry to form the comparison cohorts. First, a group consisting of women with T2DM and subsequent biopsy-confirmed BC in the period 2005–2013 was formed. Women from this group with <1 year between T2DM and BC were excluded to avoid the adaptation period to T2DM. We also excluded women without glycosylated hemoglobin (Hb1Ac) data. Second, a matched group for comparison was formed of women with T2DM but without BC during the study period. Matching was 1:2 and performed according to place of residence (same reference hospital and PHC center), age at T2DM diagnosis, and duration of T2DM. ‘Non-exposed’ women were considered if the three matching criteria were met simultaneously. Women in the T2DM+BC group had follow-ups for up to five years after a BC diagnosis. Women in the T2DM group were monitored for a time equivalent to that of their exposed partner. The BC date with which they were paired was considered a reference.

**Figure 1:**
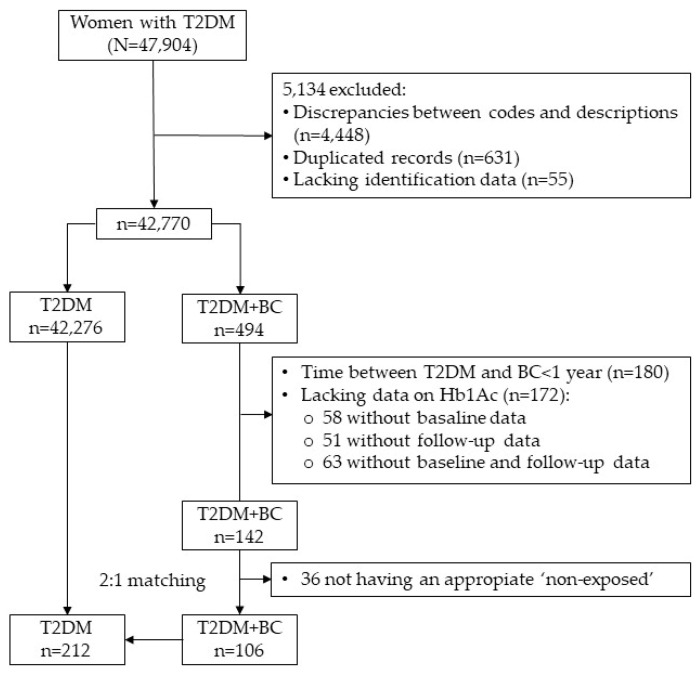
Study flowchart.

The final study sample consisted of 318 women, namely 106 women in the T2DM+BC group and 212 women in the T2DM group. The process of participant selection and assigning the study groups is shown in (Figure 1).

### 2.2. Ethical Considerations

This study was performed in accordance with the 1964 Helsinki Declaration and its later amendments or comparable ethical standards. These data were handled anonymously and confidentially. Given the nature of the study, individual informed consent was not required. This study was approved by the Board of Directors of the Public Health Service and Clinical Research Ethics Committee of Asturias (Spain) (ref. 137/17).

### 2.3. Study Variables

All data were collected by means of an exhaustive individual review of each PHC electronic medical record.

This study considered several objective parameters of cardiometabolic health measures under standardized conditions during regular PHC visits. Specifically, we collected body mass index (BMI) by dividing weight (kg) by the square of height (m), systolic and diastolic blood pressure (SBP and DBP, mm Hg), total cholesterol (mg/dL), low-density and high-density lipoprotein (LDL and HDL, mg/dL) levels, and two indicators of glycemic control: fasting blood glucose (mg/dL) and Hb1Ac (%, mmol/mol). Two quantitative values for each parameter were considered, one prior to BC diagnosis (or an equivalent date in unexposed women) and one after. As the clinical records were incomplete, any value reflected in the electronic medical records for the two years prior to diagnosis and the five years post-diagnosis was considered. Additionally, the following normative cut-off points were used to categorize the parameters according to good or poor control, with poor control corresponding to values BMI ≥ 30, SBP ≥ 130 mm Hg, DBP ≥ 80 mm Hg, total cholesterol ≥ 200 mg/dL, LDL ≥ 100 mg/dL, HDL < 40 mg/dL, fasting blood glucose ≥ 126 mg/dL, and Hb1Ac ≥ 6.5% (48 mmol/mol) [18,20,21].

Medical diagnoses after BC (or an equivalent date in unexposed women) of the following microvascular complications associated with T2DM were also collected: retinopathy, diabetic foot ulcers, nephropathy, neuropathy, and hyperuricemia.

Information was also obtained on some sociodemographic and healthcare-related variables, including age, residential area (urban: >50,000 inhabitants, suburban: 5000–49,999 inhabitants, rural: <5000 inhabitants), age at time of T2DM diagnosis, and mean number of visits per year to the PHC medical and nursing office (before and after BC or an equivalent date). Electronic medical records also contained self-reported information on health behaviors, including adherence to treatment and self-monitoring of capillary blood glucose at home. In addition, PHC providers reported whether women received counseling for alcohol, smoking, diet, or physical activity after T2DM diagnosis, suggesting the need for behavioral changes. However, the electronic medical records lacked information on actual adherence to these lifestyle behaviors.

Finally, the following chronic conditions diagnosed by a physician at the start of follow-up were also considered: cardiovascular disease (ischemic heart disease, angina pectoris, acute myocardial infarction, venous and vascular insufficiency, or chronic ischemic heart disease), stroke, arterial hypertension, dyslipidemia, mental illness (depression or anxiety), and osteoporosis.

### 2.4. Statistical Analysis

To study the differences between the two groups, Pearson’s chi-square tests and Student’s *t*-tests for independent samples were used depending on the qualitative or quantitative nature of the variables. Then, paired *t*-tests were used to assess differences in the mean of cardiometabolic parameters within individuals from baseline to follow-up in both groups.

In addition, compliance with each criterion for adequate cardiometabolic control during follow-up was estimated by conditional logistic regression using place of residence, age at T2DM diagnosis, and time of T2DM evolution as matching variables. Odds ratios (ORs) and their 95% confidence intervals (CIs), representing the probability of having cardiometabolic parameters outside the normal range during follow-up in women with T2DM+BC, were compared to women with T2DM alone. Regression analyses were performed following two adjustment models. The first model was adjusted for the baseline value of each cardiometabolic parameter considered (i.e., the risk of having a BMI > 30 during follow-up was adjusted for baseline BMI). The second model was further adjusted for age, adherence to treatment, self-monitoring of glycemia at home, lifestyle advice given by PHC staff (alcohol, smoking, diet, and physical activity), baseline status, changes in visits to the medical and nursing office, and baseline morbidity, including prevalence of cardiovascular disease, stroke, hypertension, osteoporosis, and mental illness. In ancillary analyses, we further adjusted the results for basal BMI.

Since women with T2DM+BC had better basal glycemic control than women with T2DM alone, we stratified the analyses with basal Hb1Ac (cut-off 6.5%). In the new subset cohort of women with basal Hb1Ac < 6.5%, women with T2DM had comparable mean basal blood glucose and Hb1Ac to women with T2DM+BC; therefore, BC diagnosis should be considered as part of a complementary evaluation.

Statistical analyses were performed with STATA v.17 software (Stata Corp., College Station, TX, USA). Only *p*-values < 0.05 were considered statistically significant.

## 3. Results

There were no statistically significant differences in the baseline characteristics of women with T2DM and women with T2DM+BC (Table 1). The number of visits to PHC providers—either physicians or nurses—was also similar between the two groups and did not change significantly during follow-up (Supplementary Figure S1).

Table 2 shows significant improvements in women with T2DM during follow-up in BMI, DBP, total cholesterol, HDL cholesterol, and fasting blood glucose. In women with T2DM+BC, there was an improvement in the same indicators, except for basal blood glucose, which worsened during follow-up. In this group, Hb1Ac also worsened from 6.74% (50 mmol/mol) to 7.17% (55 mmol/mol) (*p*-value = 0.009). Worsening of glycemic control after BC diagnosis particularly affected women with basal Hb1Ac < 6.5% (Supplementary Table S1).

When these indicators are modeled dichotomously (good control vs. poor control), women with T2DM+BC had an increased risk of having a basal glycemia ≥126 mg/dL (fully adjusted OR: 1.85; 95%CI: 1.01–3.41) and a Hb1Ac ≥ 6.5% (48 mmol/mol) (fully adjusted OR: 2.48; 95%CI: 1.23–4.99) (Table 3). Further adjustment for basal IMC did not materially change the results.

There were no differences between groups for the incidence of T2DM complications (Table 4).

## 4. Discussion

According to our retrospective cohort study results, in a sample of women with T2DM, subsequent BC diagnosis triggered a worsening of glycemic control measured in terms of fasting blood glucose and Hb1Ac. However, the incidence of T2DM complications was similar in women with T2DM and BC compared to women with T2DM alone.

To date, the evolution of glycemic control and other cardiometabolic health indicators in women with T2DM following a BC diagnosis has been poorly studied. Calip et al. retrospectively followed a sample of 509 women with T2DM from the United States for one year prior to BC diagnosis and three years after [27]. According to their results, women with T2DM experienced a significant worsening in HbA1c numbers [27]. The mean HbA1c increased from 6.96% before BC diagnosis to 7.42% two years later (*p* < 0.001) [27]. However, this study lacked a group of women diagnosed with T2DM alone for comparison [27]. In women with T2DM+BC, our findings are virtually identical (+0.43 percentage point increase in HbA1c). Our study also provided results for a matched comparison group that did not change during follow-up. Griffiths et al. conducted a well-designed cohort study using UK PHC data, which included 3194 women with T2DM propensity scores matched with 1036 women with T2DM+BC [28]. After a five-year follow-up, they also found that women with T2DM+BC were less likely to maintain HbA1c below the optimal level (fully adjusted OR: 0.72; 95% CI: 0.61–0.85), considered to be 59 mmol/mol, which is equivalent to 7.5% [28]. In addition, this study had a large sample. The analyses were adjusted for certain relevant lifestyle variables that were not considered, such as alcohol and tobacco consumption [28]. Conversely, our study adjusted for variables related to PHC, especially the baseline number and changes in the number of visits per year to primary care physicians and nurses, which are key to glycemic control. Additionally, we found that fasting blood glucose numbers worsened. Although a weaker indicator of glycemic control, fasting glucose numbers are useful because they are measured more frequently than HbA1c during routine clinical care. Therefore, our results provide consistency with the few previous studies available, and consistency is considered an important criterion of causality [27,28].

In our sample, it is striking that women with T2DM+BC had a better baseline lipid profile and glycemic control than women with T2DM alone. Based on the mechanisms underlying the relationship between T2DM and BC, it would be expected that women with T2DM+BC would have worse baseline cardiometabolic health indicators, which would explain the onset of BC. Although our study did not capture treatment modality, women with T2DM alone were expected to have greater adherence to oral medication and, thus, better glycemic control. There is a growing body of evidence linking adherence to T2DM treatment, especially metformin, with a lower risk of BC due to its ability to reduce energy availability to cancer cells [29,30]. We did not find a satisfactory explanation for these baseline differences between the two groups, which were contrary to expectations. In any case, it seems evident that glycemic control was impaired after BC diagnosis, suggesting that tumor growth, BC treatment, and/or the psychological impact of the diagnosis have relevant effects on metabolic regulation and T2DM self-management behaviors, including adherence to pharmacologic and non-pharmacologic treatment [27,31].

Having found worse glycemic control after BC in women with T2DM makes the association between incident BC and microvascular complications of T2DM even more plausible. However, in our study, the negative impact of BC on selected complications could not be proven. There are at least three reasons why we did not find an association. First, the women in our sample were almost 80 years old; therefore, the incidence of complications may already be low in both groups. Second, for poor glycemic control to have a microvascular impact, a longer follow-up may be needed. Third, PHC in Spain is very effective in the management of highly prevalent chronic diseases and prevention of their complications. In fact, according to our data, visits to PHC physicians and nurses were similar in women with T2DM and with T2DM+BC, both before and after BC, suggesting that the protocol for T2DM control is very well established in Spain. The only study we found on this topic yielded a similar result. In a reanalysis of their cohort study of 4230 women with T2DM, Griffiths et al. found no difference in the adjusted rate of any complication between women with T2DM and women with T2DM and BC [32].

Although there is no reason to think that our findings referring to the worse glycemic control of women with T2DM+BC are due to the competing needs of BC healthcare, some simple recommendations can be made for better follow-up of women with T2DM+BC. The most important one involves improving communication between specialized care and PHC to avoid fragmentation of care for people with interacting pathologies at both levels of healthcare. In Spain, as in many other European countries, PHC physicians receive limited feedback from specialized care physicians [33]. Moreover, communication is even more difficult when it occurs between two levels of care and between two professional categories, since physicians usually care for women with BC in specialized care, and nurses oversee T2DM follow-up in PHC. A concrete recommendation to achieve this would be the development of information channels, indicators, and specific incentives to promote greater coordination among healthcare workers caring for the same individual [28]. Protocolized interventions should also be designed and implemented to maintain and improve T2DM self-management behaviors after BC [28]. These measures should also include the management of physical symptoms and psychological distress related to BC and its treatment [34]. Finally, it is necessary to convey to healthcare providers that T2DM control targets should not be lowered when BC is diagnosed. Rather, T2DM treatment and glycemic control indicators should be more rigorously monitored, with therapeutic modifications if necessary to preserve women’s cardiometabolic health.

Our study adds consistency to the scarce scientific literature on the subject and is supported by several notable strengths. It was a matched cohort study, which is useful for eliminating the confounding effect of matching variables. In addition, we could adjust for clinical factors not normally available in databases, and healthcare-related variables, such as advice given by PHC providers and the number of visits to primary care physicians and nurses. Our cohort study had some limitations, such as its retrospective nature, but its most important limitations were linked to information sources. First, the study was intended to be population-based; however, many records had to be eliminated because they lacked relevant information. Nevertheless, the percentage of elimination was similar in both groups; therefore, the bias should not be differential. A relevant factor not considered in our study was age at menopause, which is known to have the opposite effect on T2DM and BC. While early menopause increases the risk of T2DM because the menopausal transition triggers the accumulation of upper body fat and an increased incidence of insulin resistance [35], the risk of BC increases every year of menopause mainly due to longer lifetime exposure to estrogens [35]. Secondly, due to the structure of the electronic medical recording system, it was not possible to know the exact treatments used for T2DM, despite their prominent role in the evolution of glycemic control. Third, the electronic medical records lacked socioeconomic characteristics that may contribute to this association, such as the amount of income, level of education, and social support. Finally, given the older age of the women in our sample (90% aged ≥65 years) and the small sample size, our findings may not be generalizable to all women with T2DM and BC.

## 5. Conclusions

In conclusion, in our sample of women over 50 years of age—though mostly older women from Asturias (Northern Spain)—incident BC negatively impacted glycemic control in women with pre-existing T2DM, as measured by fasting blood glucose and HbA1c. However, no adverse effects on other cardiometabolic health indicators or microvascular complications associated with T2DM were found. These findings are important for BC survivors with T2DM because they suggest that their health outcomes can be further improved by addressing the complex comorbidity relationship between T2DM and BC. Prospective studies with larger samples are needed to learn about the involvement of variables not considered in our study, such as socioeconomic characteristics, lifestyle, diet, and antidiabetic and antitumor treatments.

## Figures and Tables

**Table 1:** Baseline characteristics of women with T2DM and those with T2DM+BC.

		T2DM (*n* = 212)	T2DM+BC (*n* = 106)	*p*-Value ^a^
Age, years		79.1 (9.71)	79.3 (9.59)	0.887
≥65 years, *n* (%)		23 (89.2)	10 (90.6)	0.696
Urban residential area, *n* (%)		112 (52.8)	56 (52.8)	1.000
Age at T2DM diagnosis, years		63.6 (10.1)	64.6 (9.84)	0.389
Duration of T2DM, years		15.5 (4.52)	14.6 (5.33)	0.127
Visits/year to PHC providers		23.3 (13.6)	22.4 (10.4)	0.523
Adequate therapeutic adherence, *n* (%)		172 (81.1)	88 (83.0)	0.681
Self-monitoring of blood glucose, *n* (%)		117 (55.2)	67 (63.2)	0.172
Health advice, *n* (%)				
	On alcohol	136 (64.2)	76 (71.7)	0.178
	On tobacco	128 (60.4)	72 (67.9)	0.189
	On food	200 (94.3)	97 (91.5)	0.338
	On physical activity	203 (95.8)	99 (93.4)	0.364
Morbidity, *n* (%)				
	Cardiovascular	47 (22.2)	27 (25.5)	0.511
	Stroke	11 (5.2)	7 (6.6)	0.607
	Hypertension	137 (64.6)	69 (65.1)	0.934
	Dyslipemia	81 (38.2)	45 (42.5)	0.466
	Mental	69 (32.6)	40 (37.7)	0.358
	Osteoporosis	36 (17.0)	12 (11.3)	0.134

For continuous variables, the mean (standard deviation) is shown. T2DM: diabetes mellitus type 2; BC: breast cancer; PHC: primary healthcare. ^a^ Unpaired *t*-tests and chi-square tests were used to compare women with T2DM and T2DM+BC.

**Table 2:** Mean (standard deviation) of cardiometabolic parameters before (baseline) and after (follow-up) diagnosis of BC or an equivalent repair date in women with T2DM alone.

	T2DM (*n* = 212)			T2DM+BC (*n* = 106)		
	Baseline	Follow-Up	*p*-Value ^a^	Baseline	Follow-Up	*p*-Value ^a^
BMI, kg/m^2^	31.2 (5.4)	30.5 (5.3)	<0.001	31.3 (5.3)	30.9 (4.9)	0.024
SBP, mm Hg	137 (13.8)	138 (13.7)	0.272	137 (12.5)	136 (12.2)	0.346
DBP, mm Hg	77.8 (7.4)	76.4 (6.7)	<0.001	76.5 (7.7)	74.5 (7.6)	0.001
Total cholesterol, mg/dL	207 (43.8)	194 (39.9)	<0.001	198 (39.2)	187 (35.1)	<0.001
LDL, mg/dL	123 (32.6)	112 (32.6)	<0.001	116 (35.3)	105 (32.3)	<0.001
HDL, mg/dL	52.2 (14.4)	53.3 (12.6)	0.073	55.8 (25.6)	54.6 (23.5)	0.205
Blood glucose, mg/dL	154 (54.6)	141 (39.4)	<0.001	137 (34.5)	141 (37.0)	0.072
Hb1Ac, %	7.17 (1.78)	7.15 (1.35)	0.889	6.74 (1.23)	7.17 (1.20)	0.009
Hb1Ac, mmol/mol	54.9 (19.4)	54.6 (14.8)	0.889	50.2 (13.5)	54.8 (13.1)	0.009

BMI: body mass index; T2DM: type 2 diabetes mellitus; DBP: diastolic blood pressure; LDL: low-density lipoprotein; HDL: high-density lipoprotein; Hb1Ac: glycosylated hemoglobin; SBP: systolic blood pressure; Hb1Ac: glycosylated hemoglobin. ^a^ *p*-values obtained from unpaired *t*-tests.

**Table 3:** Risk of poor control of cardiometabolic parameters during follow-up in women with T2DM+BC compared to those with T2DM alone.

		T2DM (*n* = 212)	T2DM+BC (*n* = 106)	*p*-Value
BMI ≥ 30				
	Model 1 ^a^, OR (95% CI)	1.00	1.48 (0.64–3.41)	0.357
	Model 2 ^b^, OR (95% CI)	1.00	1.06 (0.38–3.01)	0.908
SBP ≥ 130 mm Hg				
	Model 1 ^a^, OR (95% CI)	1.00	0.64 (0.35–1.19)	0.158
	Model 2 ^b^, OR (95% CI)	1.00	0.74 (0.36–1.53)	0.415
DBP ≥ 80 mm Hg				
	Model 1 ^a^, OR (95% CI)	1.00	0.60 (0.32–1.14)	0.122
	Model 2 ^b^, OR (95% CI)	1.00	0.65 (0.31–1.38)	0.261
Total cholesterol ≥ 200 mg/dL				
	Model 1 ^a^, OR (95% CI)	1.00	0.88 (0.50–1.57)	0.661
	Model 2 ^b^, OR (95% CI)	1.00	0.96 (0.49–1.86)	0.894
LDL ≥ 100 mg/dL				
	Model 1 ^a^, OR (95% CI)	1.00	1.32 (0.72–2.40)	0.365
	Model 2 ^b^, OR (95% CI)	1.00	1.40 (0.72–2.73)	0.325
HDL < 40 mg/dL				
	Model 1 ^a^, OR (95% CI)	1.00	0.72 (0.32–1.60)	0.419
	Model 2 ^b^, OR (95% CI)	1.00	0.79 (0.29–2.20)	0.656
Blood glucose ≥ 126 mg/dL				
	Model 1 ^a^, OR (95% CI)	1.00	1.82 (1.08–3.07)	0.024
	Model 2 ^b^, OR (95% CI)	1.00	1.85 (1.01–3.41)	0.048
Hb1Ac ≥ 6.5% (48 mmol/mol)				
	Model 1 ^a^, OR (95% CI)	1.00	1.98 (1.14–3.46)	0.016
	Model 2 ^b^, OR (95% CI)	1.00	2.48 (1.23–4.99)	0.011

T2DM: type 2 diabetes mellitus; BC: breast cancer; BMI: body mass index; OR: odds ratio; CI: confidence interval; SBP: systolic blood pressure; DBT: diastolic blood pressure; LDL: low-density lipoprotein; HDL: high-density lipoprotein; Hb1Ac: glycosylated hemoglobin. ^a^ Logistic regression model 1: adjusted by the baseline value of each cardiometabolic parameter under consideration. ^b^ Logistic regression model 2: additionally adjusted for age (years), residential area (urban, suburban, rural), duration of T2DM (years), adherence to treatment, self-monitoring of blood glucose, lifestyle advice (alcohol, tobacco, diet, and physical activity), baseline status, changes in physician visits, nurse visits, and baseline morbidity (cardiovascular, stroke, hypertension, osteoporosis, and mental illness).

**Table 4:** Incidence of T2DM complications after BC diagnosis or an equivalent repair date in T2DM-only women.

	T2DM (*n* = 212)	T2DM+BC (*n* = 106)	*p*-Value ^a^
Retinopathy, *n* (%)	9 (4.2)	1 (0.9)	0.112
Diabetic foot ulcer, *n* (%)	21 (9.9)	7 (6.6)	0.327
Nephropathy, *n* (%)	19 (9.0)	9 (8.5)	0.889
Neuropathy, *n* (%)	5 (2.4)	4 (3.8)	0.473
Hyperuricemia, *n* (%)	7 (3.3)	5 (4.7)	0.532

T2DM: type 2 diabetes mellitus; BC: breast cancer. ^a^ *p*-value obtained from the chi-square test.

## Data Availability

The data can be shared up on request.

## Supplementary Materials

The following supporting information can be downloaded at: https://www.mdpi.com/article/10.3390/cancers16162853/s1, Figure S1: Mean (standard deviation), number of visits/year to primary healthcare providers before (dotted line) and after (solid line) diagnosis of BC or an equivalent repair date in women with T2DM alone; Table S1: Mean (standard deviation) of cardiometabolic parameters before (baseline) and after (follow-up) diagnosis of BC or an equivalent repair date in women with T2DM alone according to basal Hb1Ac.
