# Supplementary material for: Impact of Breast Cancer on Cardiometabolic Health in Spanish Women ≥50 Years with Pre-Existing Type 2 Diabetes Mellitus
Source: Cancers (Basel). 2024 Aug 15;16(16):2853. doi: 10.3390/cancers16162853 (PMC11352853; doi:10.3390/cancers16162853)
Supplement: Supplementary file 1 [file cancers-16-02853-s001.zip › cancers-3109384-supplementary.pdf]

Supplementary Table S1. Mean (standard deviation) of cardiometabolic parameters before (baseline) and after (follow-up) diagnosis of BC or equivalent repair date in women with T2DM alone according to basal Hb1Ac

	T2DM (n=212)			T2DM+BC (n=106)		
	Baseline	Follow-up	p-value	Baseline	Follow-up	p-value
Basal Hb1Ac<6.5%						
Blood glucose, mg/dl	125 (20.4)	127 (66.7)	0.962	124 (21.3)	133 (31.3)	0.027
Hb1Ac, %	5.81 (0.48)	6.43 (0.78)	<0.001	5.90 (0.39)	6.68 (0.84)	<0.001
Basal Hb1Ac≥6.5%						
Blood glucose, mg/dl	176 (62.1)	150 (45.5)	<0.001	150 (40.3)	150 (45.5)	0.945
Hb1Ac, %	8.23 (1.70)	7.71 (1.44)	0.020	7.62 (1.20)	7.68 (1.30)	0.689

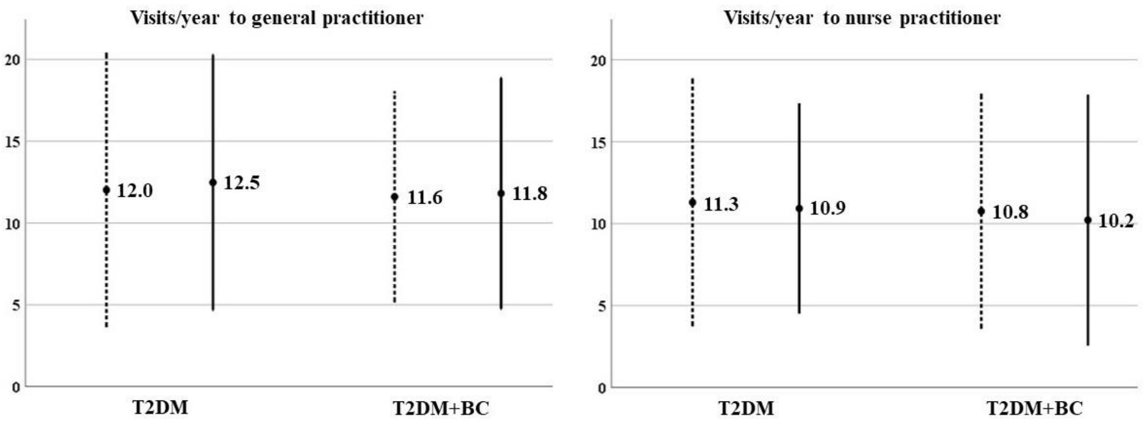

Supplementary Figure S1. Mean (standard deviation), number of visits/year to primary healthcare providers before (dotted line) and after (solid line) diagnosis of BC or equivalent repair date in women with T2DM alone.
